# Current views on sustainability in urology: findings from the North Central Section of American Urological Association membership survey

**DOI:** 10.3389/fruro.2026.1818561

**Published:** 2026-06-01

**Authors:** Chloe Shi, Susanna Wang, Ryan Steinberg, John O. DeLancy, Keow Goh, Sarah Adelstein, Pankaj Dangle, Frank C. Lin, Adam Miller, Matthew Gettman

**Affiliations:** 1 Department of Urology, Mayo Clinic, Rochester, MN, United States; 2 Department of Urology, University of Iowa Healthcare, Iowa City, IA, United States; 3 Department of Urology, The Ohio State University Wexner Medical Center, Columbus, OH, United States; 4 Department of Urology, Michigan Medicine, Ann Arbor, MI, United States; 5 Department of Urology, Rush University Medical Center, Chicago, IL, United States; 6 Division of Pediatric Urology, Riley Hospital for Children at Indiana University (IU) Health, Indianapolis, IN, United States; 7 Department of Urology, University of Wisconsin School of Medicine and Public Health, Madison, WI, United States

**Keywords:** environment, survey, sustainability, telemedicine, urology

## Abstract

**Objective:**

The United Nations defines sustainability as meeting needs of the present without compromising future generations. While often equated to “saving the planet”, sustainability is a diverse topic. Healthcare generates >5% of CO_2_ emissions worldwide, yet sustainability efforts in urology are limited. The North Central Section of the American Urological Association (NCSAUA) recently convened a Sustainability Task Force to address this topic. This study queried members on sustainability in urologic practice.

**Methods:**

Following literature review on sustainability in surgery/urology, a 19-item anonymous REDCap survey was developed and distributed via email to NCSAUA members (N = 1607). The survey assessed current best practice, institutional/personal sustainability, and demographics. Responses were compiled and summarized using descriptive statistics.

**Results:**

Survey response rate was 4.9% (79/1607). Respondents were most commonly from metropolitan areas (82%), male (71%), and in practice for 5–14 years (29%). Sustainability topics were prioritized as: (1) financial, (2) future workforce, (3) environmental. Over half (56%) reported no institutional sustainability programs or were unaware of them. Instrument (67%), computer (68%), and paper (65%) recycling were supported by a majority of respondents. However, only 28% and 29% of respondents reported current institutional use of instrument and computer recycling, respectively. Telemedicine was infrequent, with most urologists (58%) conducting 0-5% of visits virtually.

**Conclusions:**

Current institutional and urologic practice emphasis on sustainability is low. Respondents ranked highest sustainability priorities as financial and future workforce. Efforts to address environmental sustainability were also supported, namely reduction of disposables and waste. The findings support the development of targeted programs to advance sustainability priorities widely.

## Background

The United Nations (UN) defines sustainability as meeting the needs of the present without compromising the ability of future generations to meet their own needs (1). In the past decade, there has been increasing attention to sustainability in healthcare, as the sector has been estimated to generate 4.4% of global net greenhouse gas emissions (2). The United States, China, and the countries of the European Union account for over half of that, collectively contributing to 56% of the world’s healthcare climate footprint (2). While there are many different factors that contribute to healthcare’s carbon emission production, operating rooms (ORs) are particularly resource intensive; ORs have been identified to be three to six times more energy intensive than the rest of the hospital system and generate 28% of hospital waste (3–5). This contribution is paradoxical, as disease burden attributable to health-care related pollution in the United States has been estimated to be comparable to the years of life lost from preventable medical error deaths (6).

The impact of urological care and practice on sustainability has similarly received increasing attention. Given the procedural nature of the specialty and reliance on disposable items such as catheters, guidewires, and endoscopic equipment, urology has the potential to be a significant contributor to healthcare-related emissions and waste. Single-use catheters alone have been estimated to generate up to 85 million pound of waste every year, equivalent to more than 26,000 cars (7). A systematic review found that when comparing single-use and reusable instruments, although single-use devices produced less emissions per procedure, they generated greater overall waste compared to their reusable counterparts (8). However, differences in sterilization methods across hospitals generate inconsistencies in the actual trade-off between reusable and single-use instruments, though a potential role for hybrid approaches across urology still exists. Accordingly, recommendations aimed at reducing waste and energy consumption have emerged, such as drape-free cystoscopy, optimized fluid management systems, and surgeon-led initiatives to promote environmental considerations in their clinical practice (9). Direct calls for action exist, as more voices within the specialty grow concerned as the decade progresses (4, 10, 11). Despite this momentum, little is known about the extent to which practices have actually been implemented or the prevailing attitudes of practicing urologists towards sustainability.

The UN and academia also identify social and economic sustainability as key concepts in sustainability policy and science, often grouped with environmental concerns to represent the “three pillars of sustainability” (12). However, this remains understudied in urology and medicine, as existing studies largely focus solely on environmental sustainability, with limited exploration of these other domains. For example, a survey distributed among European Association of Urology Section Office and Working Group members focused on environmental practices exclusively, without addressing the other dimensions (13).

In 2024, the North Central Section of the American Urologic Association (NCSAUA) convened the first Sustainability Task Force in the AUA to address sustainability within urology. Herein, we aimed to characterize attitudes towards sustainability among practicing urologists and to identify areas for improvement in both personal and institutional settings. Given the urgency of adapting sustainable practices in medicine to reduce healthcare’s contribution towards the global climate crisis, we sought to gain more understanding on the current perspectives of urologists towards sustainability.

## Materials and methods

### Study population

We conducted an observational, cross-sectional survey study of NCSAUA members. All active NCSAUA members with a valid membership email were invited to participate.

### Survey design and distribution

Following a literature review on sustainability in urology and surgery, a survey assessing sustainability practices and beliefs was developed. The survey consisted of 19 items assessing current best practice, institutional and personal sustainability behaviors, and demographics. The full survey is available in the **Supplementary Appendix**. Anonymous REDCap web-surveys were distributed to all eligible NSCAUA members via email. Members received an initial survey invitation followed by two reminder emails to maximize response. Survey questions were not required to be completed, and each question had a skip option.

### Statistical analysis

Survey responses were compiled and descriptive statistics were generated. Survey results were expressed as n (number of respondents selecting each response option) and percentages (calculated from the total number of respondents who answered each question).

## Results

A total of 1607 surveys were sent out, of which 79 were completed (response rate: 4.9%). Most respondents were male (70.6%). Representation across age groups 35-44, 45-54, 55-64, and 65–74 was relatively equal (20.6% - 26.5%). Years in practice were nearly evenly divided between 5–24 years (48.5%) and 25- ≥35 years (48.5%); only 2 respondents (2.9%) had been in practice for fewer than 5 years (2.9%). The majority of respondents are in a practice setting (academic, hospital employed, or private equity owned) where they do not have direct financial control (91.3%). Baseline demographic characteristics are summarized in **Table 1**.

**Table 1:** Baseline demographics of survey respondents.

Characteristic	Number of respondents	% of respondents
Age (years)		
35-44	14	20.6%
45-54	18	26.5%
55-64	16	23.5%
65-74	14	20.6%
≥75	6	8.8%
Gender		
Female	17	25.0%
Male	48	70.6%
Prefer not to say	3	4.4%
Years in practice		
<5	2	2.9%
5-14	20	29.4%
15-24	13	19.1%
25-34	19	27.9%
≥35	14	20.6%
Type of practice		
Academic	32	47.1%
Private practice, non-private equity owned	6	8.8%
Private practice, private equity owned	8	11.8%
Hospital employed	22	32.4%
Setting of practice		
Metropolitan (population ≥50,000)	56	82.4%
Non-metropolitan (population <50,000)	9	13.2%
Small town (population=2,500-9,999)	3	4.4%

### Personal sustainability practices

When asked to rank sustainability issues in order of importance as it pertains to the future educational efforts of the NCSAUA Sustainability Committee, financial sustainability was ranked as the highest priority, followed by future workforce, environmental sustainability, work/life balance, support of current workforce, and other.

**Figure 1:**
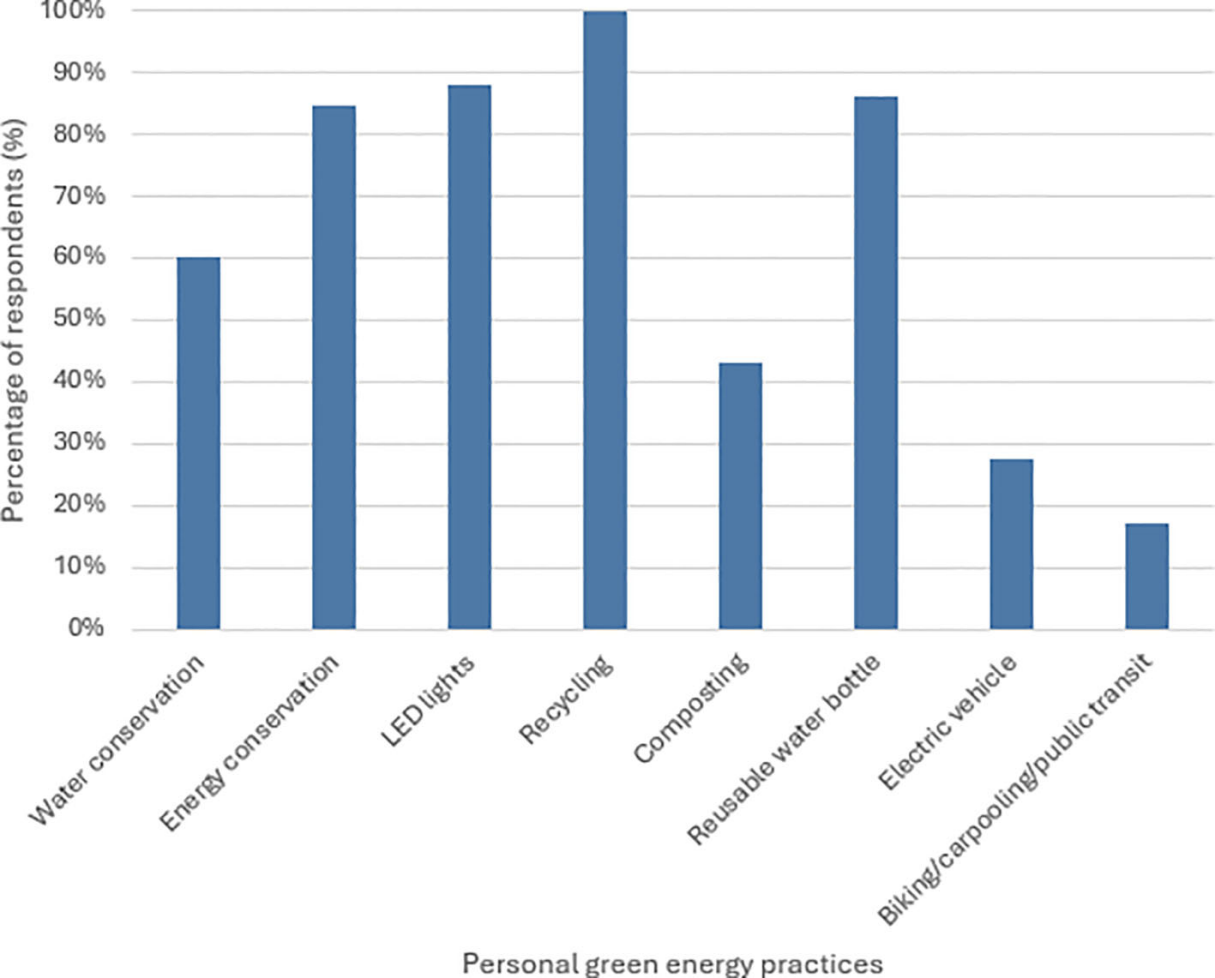
Percentage of respondents that indicated using personal green energy practices.

Sustainability practices were more prevalent in respondents’ personal lives than in their professional settings. All respondents reported participating in recycling at home. Most respondents also reported using reusable water bottles (83.8%), LED lighting (88.2%), and energy conservation (77.94%). Personal sustainability practices are summarized in **Figure 1**.

### Institutional sustainability practices

**Figure 2:**
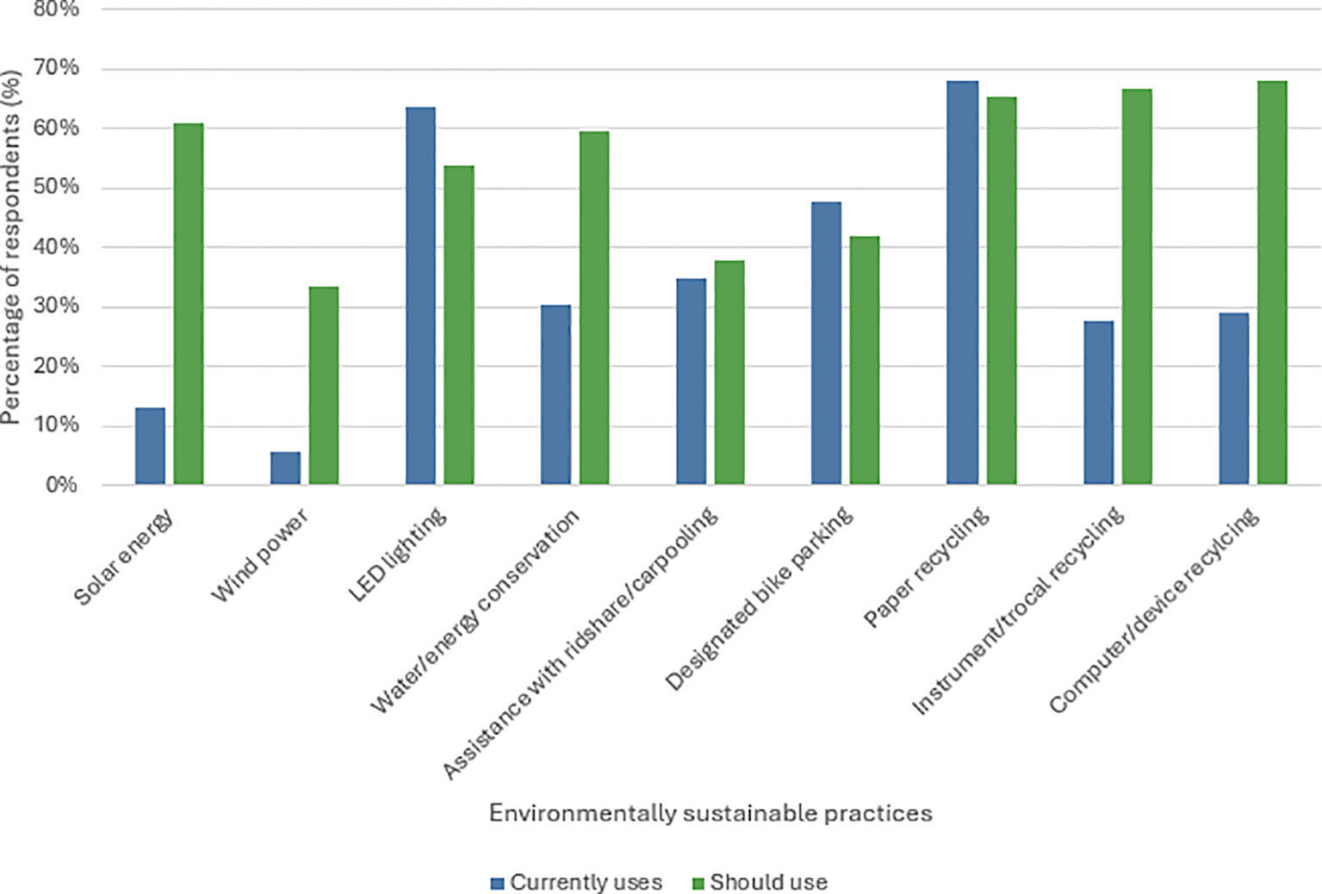
Environmentally sustainable practices that percentage of respondents know their facility currently uses vs should use.

When asked whether the facility they perform surgery at has programs or policies that support environmental sustainability in the operating room, only 12.7% of respondents said “yes, a lot”. An additional 31.7% of respondents answered “yes, a little”, while 30.4% of respondents answered “no” and 25.3% of respondents answered, “don’t know”. The most commonly reported environmentally sustainable practice at respondents’ facilities were paper recycling (68.1%) and LED lighting (63.8%). The least commonly reported practice used by facilities were wind power (5.8%) and solar energy (13.0%). Notably, respondents consistently indicated greater support for sustainability practices than their perceived institutional adoption. In six of nine listed sustainable practices, a higher proportion of respondents believed their facility should implement the practice compared with those who believed it currently did so (**Figure 2**). For example, while fewer than 30% of respondents reported their facility recycled instruments/trocars or computers/devices, over 65% of respondents believed these practices should be implemented at their facilities. Similarly, 60.9% of respondents thought their facility should use solar energy, but only 13% reported current use of solar energy.

Telemedicine use was limited among respondents: only one respondent reported conducting >40% of their appointments via telemedicine, while over half (56.5%) reported that less than 5% of their appointments were conducted virtually.

When inquired about nitrous oxide use for office or operating room procedures, most respondents (66.7%) denied use. 5.8% of respondents reported using nitrous oxide often, while the remaining 27.5% reported occasional use.

### Disposable use and waste

All respondents reported the routine use of at least one disposable item in their routine cases. The highest reported disposables used were surgical drapes and surgical gowns, with 96.2% and 94.9% of respondents reporting their routine use, respectively. A majority of respondents reported wearing disposable gowns for office-based procedures (75.4%). However, 73.9% of respondents indicated that patients were not fully draped during office cystoscopy. Laparoscopic instruments were the least commonly reported disposable, routinely used by only 13.9% of respondents. A complete list of disposables included in the survey and their reported routine use is provided in **Table 2**. For endoscopic procedures, most respondents reported opening disposables such as guidewires, ureteral catheters, and stents only as needed (86.1%). For minimally invasive and open surgeries, 43%of respondents reported that all sutures on their preference card are opened immediately at the start of the case, while 57.0% reported they were not. Over half of respondents reported that the catheters they prescribe for intermittent catheterization were intended for single use (58.0%), whereas only 10.1% reported prescribing catheters intended to be reused.

**Table 2:** Disposable item use among respondents.

Item	n	%
Flexible ureteroscopes	33	41.8%
Flexible cystoscopes	23	29.1%
Laser fibers	58	73.4%
Surgical gowns	75	94.9%
Surgical drapes	76	96.2%
Stent graspers	15	19%
Trocars	23	39.1%
Laparoscopic instruments	11	13.9%
Retractors	10	12.7%
Light handles	54	68.4%
Straps for patient positioning	38	48.1%
Fluid basins	43	54.4%

## Discussion

Advances towards environmentally sustainable practices have been highlighted as an important step in the future of urology and medicine. In this survey of practicing urologists within the NCSAUA, we shed light on the current attitudes of physicians in urology, as well as characterized what sustainable actions are being done in the status quo. Respondents ranked environmental sustainability as lower priority than financial sustainability and future workforce concerns, highlighting the multifaceted concerns currently held within the specialty. Nevertheless, respondents expressed clear support for environmental initiatives, particularly when comparing current institutional practices with those they believed should be implemented. Additionally, disposable use in the operating room and office was, unsurprisingly, common, with surgical gowns and drapes being the highest reported disposables routinely used. Urologists reported a high rate of various personal sustainability practices, such as recycling and LED lights.

While environmental sustainability has received more attention in research, our study found that practicing urologists prioritized financial and workforce concerns more highly. Growing worries about workforce shortages and burn out, particularly in rural areas, raise concerns about equitable access to urologic care for certain populations (14). Telehealth represents a particularly promising opportunity to make urologic practice more accessible and sustainable. The majority of urologists in our study reported minimal use of telehealth in their practice, with 56.5% of respondents reported having less than 5% of their office visits via telehealth. A 2025 systemic review by Hordines et al. found that all the studies they evaluated demonstrated environmental benefits from telehealth, likely due to reduced patient travel required to clinic visits (15). In addition, beyond environmental advantages, telemedicine can improve financial and economic sustainability. The Virtual Stone Clinic implemented at University Hospital Southhamptom reduced the cost per clinic appointment by 93% (16). Telemedicine has also been found to be associated with high patient satisfaction rates of over 90% and improved clinical outcomes, possible due to increased follow-up adherence and reduced time to clinical intervention (15, 17). Thus, these findings support a broader adoption of telehealth utilization among current urologists, promoting environmental and financial sustainability while utilizing a safe and effective method of clinical care. We acknowledge, however, that there are limitations associated with telehealth use, including variability of technical skills, resource availability, and acceptance of technology (18). Additionally, in the United States, telehealth implementation and use is highly sensitive to state policies, particularly those governing payment parity and cross-state practice (19). To support telehealth as a sustainable model of care, payment for telehealth services should be prioritized, and permanent infrastructure changes should be pursued to enable broader access to care, yielding both financial and environmental benefits for patients and physicians.

We found that institutional policies to support environmental sustainability in the operating room remain lacking, with over half (55.7%) of respondents reporting either an absence of policies or programs or uncertainty regarding their existence. Given the substantial contributions that operating rooms make towards hospital waste and energy consumption, this represents a critical opportunity for intervention, suggesting that more institutions should create “green” initiatives or further promote them. Prior work by Wu et al. has emphasized the value of multidisciplinary “Green Teams” comprising surgeons, anesthesiologists, nurses, and other operating room staff to drive environmentally sustainable initiatives (20). Physician leadership is critical to the effectiveness and success of these organized changes. For example, the formation of a Green Operating Room Committee by the Division of Gastrointestinal and Minimally-Invasive Surgery at the Carolinas Medical Center saved $33,000 and reduced 234.3 metric tons of CO2 emissions per year (21). These findings support the role of urologist-led initiatives in advancing meaningful environmentally conscious changes. While current institutional practice may be unsatisfactory, our study observed a motivation in physicians to improve the sustainability in their practice, an important first step towards institutional change as previously demonstrated by various sustainability teams. Additionally, while response rate was low, our sample appeared relatively representative of the 2025 American Urological Association Census of practicing urologists, with male urologists comprising the majority of the workforce (87.1%) and similar distribution across age groups (22). The low response rate may also suggest that sustainability is not yet a major focus among urologists, whereas those who are already engaged with the topic may have been more likely to participate in the survey.

Disposable use remains pervasive in urologic practice, particularly with respect to disposable gowns and drapes. Reusable surgical gowns may have a lower environmental impact than disposable gowns, with one study estimating that the benefit of producing less gowns outweighs the additional energy necessary required for laundering and reprocessing (23). However, when relating to urologic-specific equipment, such as cystoscopes and ureteroscopes, the trade-off may be less clear. While reusable scopes may generate less solid waste, they may be associated with higher emissions per use, while lack of standard sterilization processes makes these data difficult to contextualize (8). In our study, urologists identified instrument recycling as an major area for improvement, with a substantial 39% discrepancy between perceived current use and desired implementation. While we identified this as an area that is supported by urologists, more comparative studies to assess the overall environmental burden of reusable vs single-use technologies in urology should be performed.

Catheter use represents another area of ongoing debate. Only a small proportion of respondents reported prescribing catheters intended for reuse for intermittent catheterization. This seems to reflect the general attitudes and concerns of prior studies, as some suggest that reusable catheters confer unnecessary infection risk and potentially negatively affect patient quality of life (24, 25). Nonetheless, the environmental burden associated with widespread disposable catheter use is considerable and should not be overlooked, given the large volume of catheter-related waste generated annually (7).

Several limitations should be considered when interpreting our findings. First, this study was limited to surveying urologists within the North Central Section of the AUA and had a relatively low response rate, which may limit generalizability. Second, the cross-sectional design prohibits us from identifying longitudinal trends in attitudes or practices, an important factor for considering the implementation of sustainable practices. Third, while we characterize current practice and attitudes, we did not collect objective measures of outcomes such as waste production or costs. Fourth, while we draw conclusions based on cumulative survey responses, the survey design limited more advanced statistical analyses, so we were unable to assess associations between respondent characteristics and sustainability practices.

Despite these limitations, this study provides insight into current sustainability attitudes and practices among practicing urologists. While environmental sustainability was not ranked as the highest priority for urologists in the sustainability space, we identified opportunities for improvement, such as increased telehealth and reusable item use, which may align with cost savings goals with reduced operational costs and improved resource efficiency. Greater institutional engagement through sustainability committees and efforts may help align environmental goals with broader financial and workforce priorities.

## Conclusion

In this survey of NCSAUA urologists, we found that current institutional and practice-level emphasis on sustainability was limited, with financial sustainability and future workforce ranked as the highest priorities. Nonetheless, respondents expressed support for efforts to address environmental sustainability, namely through waste reduction and decreased reliance on disposable items. The findings support the development of targeted, physician-led programs to advance sustainability initiative that supports the long-term environmental, economic, and professional health of the urologic workforce.

## Data Availability

To preserve respondent anonymity, individual-level raw survey data were not made available to the study team. Conclusions were drawn from aggregated survey results provided by the survey administrators. Requests to access the datasets should be directed to Chloe Shi, chloeshi02@gmail.com.
