# Supplementary material for: Current views on sustainability in urology: findings from the North Central Section of American Urological Association membership survey
Source: Front Urol. 2026 Jun 1;6:1818561. doi: 10.3389/fruro.2026.1818561 (PMC13265346; doi:10.3389/fruro.2026.1818561)
Supplement: Supplementary file 1 [file DataSheet1.docx]

**Supplementary Appendix**

**Questions regarding surgery:**

1. When performing endoscopic procedures at your institution, most disposables (e.g. guidewires, ureteral catheters, stents) are opened for the case only as they are needed:

Yes No

1. When performing minimally invasive and open surgery at your institution, all of the suture on your preference card is opened immediately at the start of the case:

Yes No

1. Does the facility where you perform surgery have programs or policies that support environmental sustainability (e.g. educational awareness initiatives, “going green” practices, or efforts to reduce waste) particularly in the operating room?

Yes a little Yes a lot No Don’t know

4. From the list of ***disposable*** items, please check the items that you routinely use for most of your cases:

-flexible ureteroscopes

-flexible cystoscopes

-laser fibers

-surgical gowns

-surgical drapes

-stent graspers

-trocars

-laparoscopic instruments

-retractors

-light handles

-straps for patient positioning

-fluid basins

**Questions regarding your primary facility where you see patients:**

5. Check the environmentally sustainable “green” practices that you know your facility uses/endorses:

-Solar energy

-Wind power

-LED lighting

-Water/energy conservation

-Assistance with rideshare/carpooling

-Designated bike parking

-Paper recycling

-Instrument/trocar recycling

-Computer/device recycling

6. Check the environmentally sustainable “green” practices that you wish your facility has implemented:

-Solar energy

-Wind power

-LED lighting

-Water/energy conservation

-Assistance with rideshare/carpooling

-Designated bike parking

-Paper recycling

-Instrument/trocar recycling

-Computer/device recycling

7. What percentage of your appointments are telemedicine?

-0-5%

-5-20%

-20-40%

->40%

8. Does your facility use nitrous oxide (i.e. laughing gas) for office or surgery center procedures?

-Yes, often

-Yes, sometimes

-No

9. When you perform office cystoscopy is the patient fully draped?

Yes No

10. When prescribing catheters for intermittent catheterization, you prescribe?

Catheters intended for single use

Catheters intended to be reused

Selection based on individual patient factors

11. What type of gown do you wear when performing office-based procedures?

Disposable

Reusable

**Questions about you:**

12. Which term best describes your practice?

-Academic

-Private practice, non-private equity owned

-Private practice, private equity owned

-Hospital employed

13. What demographics describe the setting of your practice?

- Metropolitan areas (population ≥ 50,000)

- Non-metropolitan areas (population <50,000)

- Small town (population = 2,500-9,999)

- Rural (population < 2,500)

14. Check which “green energy” practices you use in your personal life:

-Water conservation

-Energy conservation

-LED lights

-Recycling

-Composting

-Reusable water bottle

-Electric vehicle

-Biking, carpooling, public transit

15. Please rank order the following sustainability issues in order of importance as it pertains to the future educational efforts of the NCSAUA Sustainability Committee (1= most important):

-financial sustainability

-environmental sustainability

-future workforce

-work/life balance

-support of current workforce (employee well-being, continuing medical education)

-other

16. What gender do you associate with?

-Female

-Male

-Transgender

-Non-binary

-I use a different term

-Prefer not to say

17. What is your age?

-25-34

-35-44

-45-54

-55-64

-65-74

-≥75

18. How many years have you practiced?

-<5

-5-14

-15-24

-25-34

-≥35

19. Any additional comments you would like to share with NCSAUA regarding sustainability? ____________________________________________________________________________________________________________________________________________________________________________________________________________________________________________________________________________________________________________________________________________________________________________________________________________________________________
